# Global clinical trial landscape of stem cell-based therapies for osteoarthritis: trends and translational implications

**DOI:** 10.3389/fcell.2026.1757935

**Published:** 2026-02-04

**Authors:** Xin Li, Yue Zhao, Dongsheng Guo, Chen Yan, Jian Zhang, Lin Cheng, Yuefu Dong

**Affiliations:** 1 Department of Orthopedics, The Affiliated Lianyungang Hospital of Xuzhou Medical University (The First People’s Hospital of Lianyungang), Lianyungang, Jiangsu, China; 2 Department of Nursing, Lianyungang Maternity and Child Health Hospital, Lianyungang, Jiangsu, China; 3 Department of Orthopedics, The Affiliated Hospital of Xuzhou Medical University, Xuzhou, Jiangsu, China

**Keywords:** clinical trials, mesenchymal stem cells, osteoarthritis, regenerative medicine, stem cell therapy

## Abstract

**Objective:**

Osteoarthritis (OA) is a leading cause of pain and disability worldwide, yet disease-modifying treatments remain limited. This study aimed to map the global registry landscape of interventional clinical trials of stem cell–based therapies for OA and summarize temporal, geographic, and design trends.

**Methods:**

We conducted a systematic, registry-based landscape analysis of interventional clinical trials assessing stem cell therapies for osteoarthritis. Trial records were obtained from the Informa Pharmaprojects platform. Two researchers extracted and summarized trial characteristics, including year, phase, geographic distribution, target joint, cell source or type, autologous versus allogeneic strategy, administration route, outcome measures, and trial status. We then performed a descriptive trend analysis.

**Results:**

We identified a total of 224 eligible trials. The number of trials has steadily increased over time, with broad international participation. Most studies focused on knee osteoarthritis and used intra-articular administration. Mesenchymal stem cell-based products dominated, encompassing both autologous and allogeneic approaches, with growing attention to scalable allogeneic strategies. Primary endpoints were typically patient-reported pain and functional measures, while imaging and biomarker outcomes were often secondary. Published evidence syntheses suggest potential benefits in terms of pain and function, but conclusions are frequently limited by heterogeneity, risk of bias, and relatively short follow-up durations.

**Conclusion:**

The number of stem cell–based clinical trials for osteoarthritis is increasing globally, but heterogeneity in study designs and incomplete public reporting limit reliable conclusions about efficacy. Future research should prioritize standardizing products and protocols, employing more rigorous comparators and feasible blinding, extending follow-up periods, and ensuring transparent reporting to facilitate clinical translation.

## 1 Introduction

Osteoarthritis (OA) is one of the most common joint disorders worldwide, particularly affecting older adults and imposing a substantial burden in terms of pain, disability and healthcare utilization (Wood et al., 2023; Darlow et al., 2025; Kloppenburg et al., 2025). The pathogenesis of OA is complex, typically involving the gradual degradation and loss of articular cartilage, leading to degenerative changes in the bone, synovium, and surrounding joint structures (Han et al., 2024; Jenei-Lanzl and Zaucke, 2025; Zhang et al., 2025). Typical symptoms include joint pain, stiffness, limited mobility and functional impairment, which markedly reduce patients’ quality of life and work capacity (Cho et al., 2021). Current treatment options for OA primarily include pharmacological treatments, physical therapy, and surgical interventions (Arden et al., 2021; Cho et al., 2021; Pagan et al., 2024). However, existing pharmacological, interventional and surgical treatments predominantly alleviate symptoms or delay disease progression without repairing the underlying joint damage. Long-term use is associated with adverse effects or procedure-related risks and fails to restore damaged articular cartilage effectively (Da Costa et al., 2021; Peat and Thomas, 2021; Graf et al., 2022). Therefore, developing therapeutic approaches that can repair, and regenerate articular cartilage remains a major challenge in OA management.

In recent years, stem cell therapy has emerged as a promising option and has attracted increasing attention (Hussen et al., 2024; Mei et al., 2024). Stem cells can be derived from autologous or allogeneic sources and possess self-renewal and multi-lineage differentiation capacity, making them attractive for tissue repair and regenerative medicine (Zhu et al., 2021). In OA, stem cell–based interventions are proposed to act through chondrogenic differentiation that supports cartilage repair and through paracrine secretion of growth factors and cytokines that modulate the inflammatory microenvironment and limit further cartilage damage (Zhao et al., 2021; Cai et al., 2023). Although encouraging signals have been reported in preclinical studies and small-scale clinical trials (Yan et al., 2021; Rong et al., 2024), translation into routine clinical care requires robust evidence from well-designed clinical trials. Several stem cell therapies have shown varying degrees of success in OA trials worldwide (Chen C.-F. et al., 2021; Lamo-Espinosa et al., 2021; Sadri et al., 2023), and recent research hotspots include allogeneic strategies and combination approaches with other treatments (Zhang et al., 2022; Abe et al., 2023; Hung et al., 2025).

Despite this rapid expansion, the clinical development landscape remains fragmented across heterogeneous products and trial designs, making it difficult to track how the field is evolving and where key translational bottlenecks lie. In addition, many registered trials have no publicly available results, and registry “completion” does not necessarily translate into timely peer-reviewed publication. Therefore, a systematic registry-based synthesis is needed to map the global trial portfolio and summarize temporal, geographic, and design trends.

Accordingly, in this systematic, registry-based landscape analysis, we analyzed registered interventional clinical trials of stem cell–based therapies for OA to address three objectives. First, we mapped temporal trends and geographic distribution of trial activity. Second, we characterized trial design features, including phase, target joint, administration route, cell source or type, and autologous versus allogeneic strategies, as well as commonly selected outcomes and trial status. Third, we summarized the current clinical development landscape to inform future trial design and identify areas where standardization and higher-quality evidence generation are most needed.

## 2 Materials and methods

### 2.1 Data source and search strategy

Clinical trial data were obtained from the Informa Pharmaprojects database (https://pharma.id.informa.com/), which provides structured information on global drug development programs. We searched for trials with “Disease is Autoimmune/Inflammation: Osteoarthritis” and “Drug Type is Biological > Cellular > Cell type > Stem cell.” All records registered between 1 January 2000, and 10 January 2025 were retrieved; the search was last updated on 10 January 2025.

### 2.2 Eligibility criteria and study selection

Trials were eligible if they enrolled human participants with osteoarthritis as a primary indication, investigated a stem cell or stem cell–derived product as a therapeutic intervention, and were registered as interventional studies in any clinical phase. Preclinical studies, observational designs, trials in which osteoarthritis was only a secondary indication, and records without a stem cell–based intervention were excluded. Duplicates, registry updates, and obvious extensions were merged using trial identifiers and sponsor information. For clarity, the inclusion and exclusion criteria are summarized in Table 1. After screening, 224 unique clinical trials were included in the final dataset.

**TABLE 1:** Inclusion and exclusion criteria for trial selection.

Domain	Inclusion criteria	Exclusion criteria
Population	Human participants	Non-human studies
Condition/Indication	Osteoarthritis as the primary indication	Osteoarthritis only as a secondary indication
Intervention	Stem cell or stem cell–derived product used as a therapeutic intervention	Records without a stem cell–based intervention
Study design	Interventional clinical trials	Observational designs
Trial phase	Any clinical phase	Preclinical studies
Record handling	Unique trials after de-duplication	Duplicates, registry updates, obvious extensions counted as separate trials
Final inclusion	Trials meeting all criteria above	Trials failing any criterion above

### 2.3 Data extraction and variables

Two investigators independently extracted data from the Informa entries using a standardized form and resolved discrepancies by discussion. The following information was collected: trial title, year of initiation, phase, planned or actual sample size, study design, recruitment status, sponsor type, and country, which was further grouped into geographic regions. Stem cell–related variables included cell source, autologous versus allogeneic use, administration route and regimen, and major combination strategies. Outcomes were classified as primary or secondary and grouped into patient-reported measures, functional scores, imaging endpoints, quality-of-life instruments, and safety endpoints. Biomarkers reported as eligibility criteria or outcomes were categorized into inflammatory markers, cartilage or tissue turnover markers, imaging biomarkers, and routine laboratory parameters. Trial status was coded as planned, ongoing, completed, terminated, discontinued, or not initiated.

### 2.4 Statistical analysis

**FIGURE 1:**
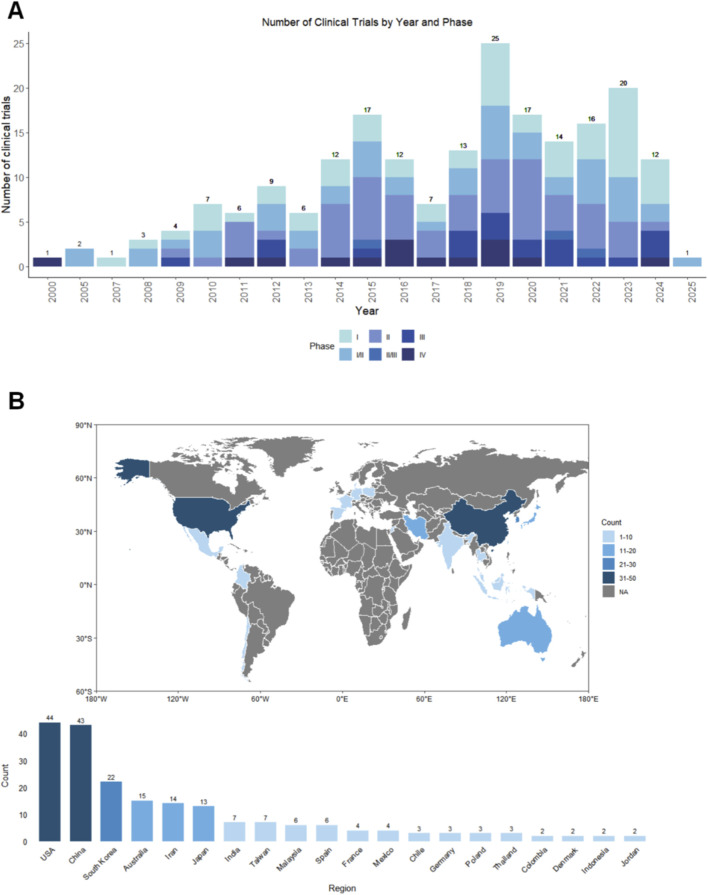
**(A)** Temporal distribution of clinical trials by year and phase. **(B)** Global distribution and regional breakdown of clinical trials by region.

**FIGURE 2:**
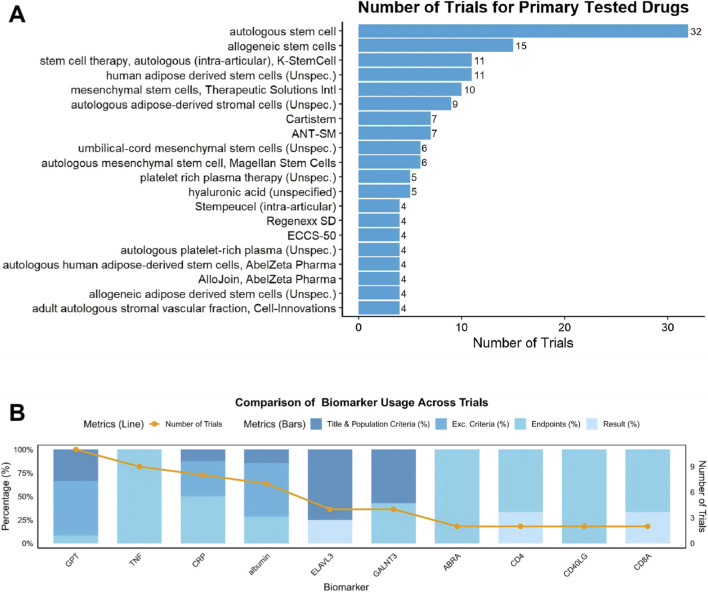
**(A)** Distribution of clinical trials by primary treated drugs. **(B)** Comparison of oncology biomarker usage across clinical trials, categorized by biomarker type and associated trial percentages.

**FIGURE 3:**
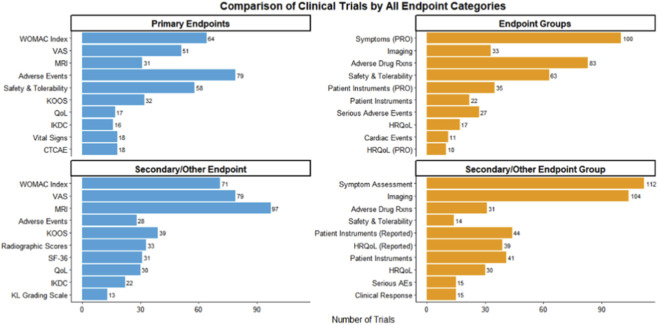
Comparison of clinical trials by endpoint categories.

**FIGURE 4:**
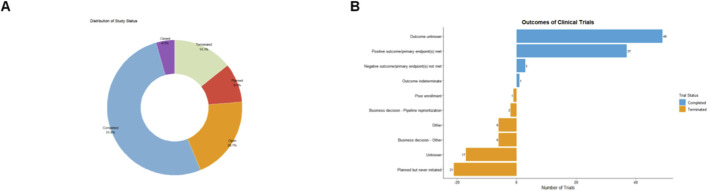
Distribution and outcomes of clinical trials. **(A)** Proportion of clinical trial data categories as shown in the pie chart. **(B)** Number of clinical trials by outcome category.

All extracted data were entered into a spreadsheet and analyzed descriptively using R (R Foundation for Statistical Computing, Vienna, Austria). Categorical variables were summarized as counts and percentages. Temporal trends were described by the annual number of newly registered trials and their phase distribution. Geographic patterns, stem cell product characteristics, biomarker use, endpoint selection, and trial status were visualized with bar and line charts corresponding to Figures 1–4. Because of the heterogeneity of designs and the registry-level nature of the data, no formal between-trial comparative statistics or meta-analysis was performed.

### 2.5 Ethical considerations

This study used de-identified, trial-level information from a commercial clinical trial database without accessing individual patient data. According to institutional policy and local regulations, separate approval from an ethics committee or institutional review board was not required.

## 3 Results

### 3.1 Overall characteristics of registered clinical trials

A total of 224 interventional clinical trials evaluating stem cell–based therapies for osteoarthritis were identified in the Informa database between 2000 and 2025. These trials collectively span early-phase feasibility and safety studies through to Phase III efficacy trials and encompass both autologous and allogeneic cell products administered predominantly by intra-articular injection. The overall dataset captures a broad spectrum of trial designs, cell sources, dosing regimens, and outcome measures and forms the basis for the descriptive analyses summarized in Figures 1–4.

### 3.2 Temporal trends and geographic distribution

The annual number of registered trials remained low and relatively stable from 2000 to 2010, followed by a marked expansion in activity from 2010 onwards. As shown in Figure 1A, the number of new trials increased steadily during the past decade, with distinct peaks in 2018 and 2021. These peaks coincided with the wider clinical adoption of mesenchymal stem cell (MSC) products and growing interest in regenerative strategies for osteoarthritis. In addition to the overall rise in trial numbers, there was a gradual shift in trial phase composition over time: early work was dominated by Phase I and II studies focused on safety and preliminary efficacy, whereas the increasing proportion of Phase III trials has been observed predominantly over the past decade, reflecting a transition toward larger studies designed to assess longer-term outcomes in more heterogeneous patient populations.

Geographically, clinical research on stem cell therapy for osteoarthritis is concentrated in a limited number of countries (Figure 1B). The United States and China account for the largest share of registered trials (44 and 43 trials, respectively), followed by South Korea (22 trials) and Australia (15 trials). Additional studies were conducted in Japan, Iran, India and several European countries, although typically with smaller trial counts. Overall, North America and the Asia–Pacific region constitute the main hubs of clinical activity, reflecting both the availability of research infrastructure and the presence of regulatory pathways that support early clinical translation of cell-based therapies.

### 3.3 Stem cell products and biomarker use

Across the 224 trials, a wide range of stem cell products and delivery strategies were investigated (Figure 2A). Autologous stem cell therapies were the most frequently studied, representing 38% of all trials. These products were commonly derived from the patient’s own bone marrow, adipose tissue or peripheral blood and were reinjected into the affected joint. Allogeneic products—obtained from unrelated donors—constituted a substantial and growing proportion of studies, particularly in more recent years. Allogeneic MSCs from bone marrow, adipose tissue and umbilical cord sources were prominent among these products, reflecting their scalability and the possibility of off-the-shelf use.

In addition to stand-alone cell therapies, several trials evaluated combination approaches, such as MSCs administered together with platelet-rich plasma or other biological agents. These protocols were designed to leverage potential synergistic effects on cartilage repair, inflammation control and overall joint function.

Biomarkers were widely incorporated into trial designs, either as eligibility criteria or as exploratory endpoints (Figure 2B). Frequently used markers included indices of inflammation and tissue injury such as C-reactive protein, tumor necrosis factor and liver enzymes. These biomarkers were used to characterize baseline disease activity, monitor treatment response and, in some studies, define specific patient subgroups. Their recurrent use across trials underlines the central role of inflammatory and degenerative pathways in osteoarthritis and highlights ongoing efforts to link biological changes with clinical outcomes.

### 3.4 Primary and secondary endpoints

Patient-reported outcomes constituted the core primary endpoints in most trials investigating stem cell therapy for osteoarthritis. As illustrated in Figure 3, the Western Ontario and McMaster Universities Osteoarthritis Index (WOMAC) and the visual analog scale (VAS) for pain were the most commonly selected primary measures. WOMAC was used to assess pain, stiffness and physical function, whereas VAS captured the intensity of pain perceived by patients. Other primary endpoints used less frequently included additional pain or function scales and composite indices of symptom burden.

Secondary endpoints provided complementary information on structural changes, quality of life and safety. Radiological evaluation was prominent: 97 trials incorporated magnetic resonance imaging (MRI) as a secondary endpoint, reflecting its ability to assess cartilage integrity, synovial inflammation and other soft tissue features relevant to osteoarthritis progression. Conventional radiography was also used to monitor joint space and bony changes over time.

Functional assessment tools such as the Lysholm Knee Score and the International Knee Documentation Committee (IKDC) score were used as secondary outcomes in a subset of studies, providing additional detail on knee stability, activity limitations and sport-related function. Health-related quality-of-life instruments were employed to capture the broader impact of treatment on daily living.

Safety was formally assessed in many trials. Thirty-one studies explicitly recorded adverse drug reactions as secondary endpoints, allowing systematic evaluation of local and systemic events after intra-articular stem cell administration. Across these studies, the most frequently reported adverse events were transient injection-site pain, swelling and mild inflammatory reactions, with serious treatment-related complications rarely described.

### 3.5 Trial status and reported outcomes

The status distribution of the 224 trials is summarized in Figure 4. Overall, 51.8% of trials had been completed at the time of data extraction, while 20.1% were ongoing and 9.4% remained in the planning or start-up stage. In contrast, 14.3% of trials had been terminated, and 4.5% were discontinued prematurely before reaching their planned endpoints. In addition, 21 trials were registered but never initiated, underscoring the practical and regulatory hurdles that can impede the progression of stem cell products from concept to clinical evaluation.

Among completed trials, the majority reported favorable safety and tolerability profiles. Intra-articular stem cell injections were generally well tolerated, with most adverse events being mild and self-limiting local reactions. Although dosing regimens, cell sources and follow-up durations varied widely, there was no consistent signal suggesting serious treatment-related toxicity.

Clinical efficacy outcomes were more heterogeneous but overall encouraging. A substantial proportion of completed trials reported statistically significant improvements in primary patient-reported endpoints such as WOMAC and VAS compared with baseline or control conditions, often accompanied by gains in functional scores and quality-of-life measures. In trials that incorporated advanced imaging, MRI frequently demonstrated stabilization or partial restoration of cartilage structure in parallel with symptom improvement. Taken together, these findings indicate that, within the current landscape of registered clinical studies, stem cell–based therapies for osteoarthritis have shown a generally favorable safety profile and a promising, though still evolving, pattern of clinical benefit.

## 4 Discussion

### 4.1 Trends and geographic distribution

Over the past 2 decades, trial activity has accelerated, particularly after 2010, reflecting not only advances in stem cell science but also a set of translational drivers that make OA an attractive and feasible target for clinical development. These include the large unmet clinical need in a highly prevalent chronic disease, persistent limitations of existing symptom-focused treatments, increasing industrialization of cell products with greater sponsor participation, and the practicality of regulatory and ethics pathways for intra-articular interventions in many settings. In addition, many trials can generate interpretable short-term signals using commonly adopted symptoms and function endpoints, which lowers operational barriers and may further encourage registrations. The observed shift from smaller early-phase studies to larger late-phase programs therefore primarily reflects feasibility and early signals that warrant further testing rather than definitive confirmation of effectiveness. Consequently, growth in registrations should be interpreted as increased research momentum and design experimentation, not as evidence that efficacy has matured at the same pace.

Stem cell therapies for OA have been studied in several countries, with trial activity concentrated in a limited number of regions. Beyond general research infrastructure and funding, this concentration is plausibly driven by differences in cell manufacturing capacity and compliance systems, the presence of commercial developers and established clinical trial networks, and variability in national regulatory requirements that shape how readily cell-based interventions can enter clinical testing. These structural factors influence not only how many trials are registered but also how interventions are manufactured, delivered, and monitored across sites. A key implication is that geographic clustering can amplify cross-trial differences in patient mix, background care, and implementation practices, reducing comparability and making it harder for a growing trial count to translate into clearer, more generalizable conclusions.

However, the global distribution of stem cell trials for OA also underscores a central translational challenge: heterogeneity across regions can directly contribute to inconsistent effect estimates and uncertainty when evidence is synthesized. Differences in eligibility criteria, OA phenotyping and severity mix, comparator selection and blinding execution, co-interventions, imaging acquisition and scoring protocols, and follow-up duration can all widen between-study variability and complicate interpretation of symptom and structural outcomes. As a result, the field’s bottleneck is not simply the number of trials but whether trials converge on sufficiently harmonized designs and reporting standards to permit robust cross-study comparison and credible pooled inference. In this context, standardization efforts are essential to improve evidence coherence and strengthen the robustness of conclusions drawn across diverse settings (Park et al., 2021; Hirai et al., 2023).

By fostering greater global cooperation and standardizing trial methodologies, the stem cell research community may improve cross-study comparability and reduce avoidable sources of heterogeneity; however, a structural translational gap can still persist in which trial volume grows faster than reproducible, clinically meaningful efficacy is established. This gap is largely driven by variability in product characterization and manufacturing, inconsistent comparator rigor and blinding, limited long-term follow-up for a chronic disease, and delays or incompleteness in results dissemination. Therefore, international collaboration should prioritize not only recruitment and geographic expansion but also harmonized product reporting, rigorous late-phase designs with appropriate comparators, longer follow-up, and transparent publication of complete results to convert research activity into a more definitive evidence base.

### 4.2 Stem cell applications and biomarkers

In the included trials, most interventions used either autologous or allogeneic products, with autologous approaches still predominating overall. This pattern is likely driven less by efficacy considerations and more by early translational feasibility, including regulatory and ethical acceptability, local access to harvesting procedures, and the practicality of implementing individualized preparations within existing clinical workflows (Li et al., 2021). In parallel, the increasing use of allogeneic products appears to reflect a shift toward scalability and operational efficiency, including lower per-dose cost at scale, batch manufacturing, improved logistics, and the potential for more consistent supply and quality control within organized development programs (Copp et al., 2023). Across these sourcing strategies, MSC- and ADSC-based approaches remain common in the registry landscape (Boulestreau et al., 2021; Tevlin et al., 2022; Xiang et al., 2022), consistent with their broad adoption in regenerative pipelines and the expectation that paracrine and immunomodulatory actions may be relevant to OA symptom modulation. However, registry categorization by cell “type” provides only a coarse description of the intervention, because manufacturing conditions, product characterization, dosing schedules, and co-interventions can vary substantially and may contribute to heterogeneous clinical signals. A growing proportion of trials also combine cell-based interventions with adjunctive biological strategies such as PRP, which may be interpreted as an attempt to amplify therapeutic signals or reduce inter-individual variability in response; at the same time, such combinations introduce co-intervention complexity and can further complicate causal attribution and cross-trial comparability, thereby widening the gap between trial volume and interpretable evidence (Zhao et al., 2022).

Biomarkers may add value for baseline characterization, safety context, and exploratory stratification, but their current use in OA stem cell trials remains largely non-specific and hypothesis-generating rather than confirmatory. In our dataset, laboratory measures including liver enzymes and inflammatory markers such as GPT, TNF, and CRP were commonly incorporated, often as screening variables or general indicators of systemic status (Ansari et al., 2020; Van Berkel et al., 2022; Zheng et al., 2023). While these measures can help contextualize comorbidity burden and inflammatory milieu, they are not OA-specific and are not validated surrogate endpoints for OA progression or treatment response. Consequently, their frequent inclusion more likely reflects an active search for stratification signals than an established ability to predict or explain efficacy. Future trials would benefit from pre-specified biomarker hypotheses, harmonized collection and reporting procedures, and explicit linkage of biomarker readouts to imaging changes and clinically meaningful responder definitions, so that biomarker use strengthens mechanistic interpretation and reduces heterogeneity rather than adding additional layers of non-comparable exploratory data.

### 4.3 Primary and secondary endpoints

In clinical trials investigating stem cell therapy for OA, primary endpoints typically emphasize symptomatic relief and functional improvement, reflecting both the clinical priorities of patients and the practical realities of trial conduct. Most studies select patient-reported outcomes such as WOMAC and VAS as primary endpoints, not only because they capture pain and disability—the dominant drivers of OA burden—but also because they are feasible to implement across sites, sensitive to short-term change, and less resource-intensive than structural endpoints. This design choice can facilitate recruitment and follow-up and may help trials detect early signals of benefit within relatively short observation windows. However, while these measures are appropriate for evaluating symptomatic benefit, they are also susceptible to contextual effects, co-interventions, and variability in background care, and they do not by themselves establish durable structural modification or disease modification.

From a clinical perspective, these trials sit alongside established symptomatic OA treatments such as oral anti-inflammatory analgesics and intra-articular injectables including corticosteroids and hyaluronic acid (Kjeken et al., 2025; Sherman et al., 2025). Conventional therapies have comparatively mature evidence bases and standardized use in routine care, whereas stem cell–based approaches are often positioned as regenerative or potentially disease-modifying strategies. Notably, many stem cell trials rely on the same symptom-driven endpoints commonly used in conventional-treatment studies, which makes it difficult to determine whether observed improvements represent incremental benefit beyond placebo effects, injection-related effects, or symptomatic gains achievable with standard care, particularly when active-comparator designs are limited and follow-up is relatively short. As a result, comparative effectiveness relative to conventional therapies remains insufficiently defined, reinforcing the need for more rigorous designs with appropriate active comparators, standardized background care, longer follow-up, and structural endpoints to clarify translational value.

Secondary endpoints are commonly used to broaden interpretation beyond symptoms, including structural assessment, longer-term outcomes, and safety. Radiological evaluations such as MRI and X-rays are frequently incorporated, with many trials positioning MRI as a secondary endpoint, which is informative in itself: it suggests that structural regeneration and disease modification remain hypotheses under evaluation rather than consistently demonstrated effects. MRI can provide objective information on cartilage status, synovitis, and other joint tissues and is therefore valuable for mechanistic plausibility and structural signal detection (Roemer et al., 2022; Hayashi et al., 2025). At the same time, cross-trial comparability of imaging outcomes can be limited by differences in acquisition protocols, scoring systems, and analytic approaches, which may contribute to heterogeneous structural findings and complicate evidence synthesis. Other secondary endpoints, including QoL measures and functional scales such as Lysholm and IKDC, extend the outcome framework to broader health impact and activity limitation, but they also increase outcome heterogeneity across studies when instruments and timing vary. Safety monitoring, often captured through adverse reactions as secondary endpoints, remains essential for interpreting benefit–risk balance, yet safety ascertainment and reporting can be inconsistent when follow-up is short or dissemination is incomplete. Taken together, current endpoint selection supports a multidimensional assessment of symptom, function, structure, and safety, but interpretation remains constrained by heterogeneity in endpoint definitions, measurement schedules, and reporting practices.

### 4.4 Clinical trial status and completion rates

Among registered trials of stem cell–based therapies for OA, a substantial proportion have reached completed status, indicating that recruitment and follow-up were finished as planned. However, completion recorded in a registry should not be interpreted as evidence maturity, because it does not necessarily imply that results have been posted, peer-reviewed, or reported with sufficient methodological rigor to support clinical effectiveness claims. Accordingly, completed and ongoing registrations are best viewed as indicators of research feasibility, operational throughput, and sustained development activity rather than direct proxies for treatment efficacy. This distinction is critical because a growing number of completed trials can coexist with persistent uncertainty when products, comparators, and outcome time-points vary widely and when results dissemination is incomplete or delayed.

In addition to completed and ongoing trials, a proportion of studies remain in planning stages, while others have been terminated, discontinued, or never initiated. These patterns are informative for understanding translational friction in the field. Trial termination or discontinuation may reflect not only funding and recruitment barriers but also protocol impracticality, evolving regulatory requirements, manufacturing or quality-control constraints, or insufficient early signals to justify continuation. Similarly, trials registered but never initiated suggest that the path from preclinical promise to executable clinical programs remains vulnerable to operational and regulatory constraints. Importantly, these dynamics can generate visibility and reporting biases in registry-based landscapes, because completed registrations without public results and discontinued programs without transparent outcome reporting can distort the apparent strength and coherence of the evidence base. Consequently, efficacy signals remain difficult to interpret without systematic linkage to peer-reviewed publications and risk-of-bias appraisal, and observed patterns in adverse events and outcomes should be interpreted cautiously when reporting is incomplete.

Overall, while many trials have been completed or are ongoing, the translational gap between trial volume and proven, durable clinical efficacy remains substantial. Therefore, registry completion and trial activity should be interpreted primarily as indicators of research momentum and design patterns rather than definitive evidence of effectiveness.

### 4.5 Advances and safety of stem cell therapy for osteoarthritis

In recent years, substantial progress has been made in stem cell therapy for OA, with multiple clinical studies reporting encouraging signals for symptom improvement, reflecting the rapid development of regenerative medicine in joint disease management (Copp et al., 2023; Yin et al., 2023; Cui et al., 2024). Stem cell–based interventions, particularly those derived from the umbilical cord, adipose tissue, and bone marrow, have been explored as minimally invasive options for OA management (Cao et al., 2023; Lagneau et al., 2023; Lin et al., 2025). The predominance of intra-articular delivery and these product sources likely reflect translational practicality, including procedural feasibility, a localized delivery rationale for targeting the joint microenvironment, and comparatively accessible regulatory and cost pathways for clinical testing. However, this feasibility can also increase registrations and trial throughput without necessarily indicating that efficacy evidence has converged across products and settings. Their immunomodulatory and paracrine effects are hypothesized to help mitigate local inflammation and support cartilage homeostasis, although the relative contributions of different mechanisms remain under investigation (Xu et al., 2023).

To provide an external quality check on whether registry-level growth in trial activity aligns with the peer-reviewed evidence base, we compared these trends with recent peer-reviewed systematic reviews and meta-analyses (Sadeghirad et al., 2024; Bensa et al., 2025; Cao et al., 2025; Whittle et al., 2025). Overall, these syntheses generally suggest that intra-articular MSC administration may be associated with improvements in pain and function outcomes, frequently assessed using WOMAC and VAS, compared with control comparators; however, the certainty of evidence is often limited by substantial clinical and methodological heterogeneity, variability in cell products and dosing regimens, short follow-up, and risk of bias across trials. Importantly, pooled effect sizes are frequently modest and, in some analyses, may approach or fall below commonly used minimal clinically important difference thresholds, underscoring that an expanding trial pipeline does not necessarily translate into reproducible, clinically meaningful efficacy (Sadeghirad et al., 2024; Whittle et al., 2025). This attenuation of pooled effects is plausibly driven by dispersion in product characterization and dose–schedule choices, variation in comparator rigor and blinding, short follow-up windows, and inconsistent endpoint definitions and assessment time-points across trials. Accordingly, our registry-based findings should be interpreted as describing research activity and design patterns rather than as direct evidence of effectiveness, and efficacy inferences should primarily rely on peer-reviewed syntheses and well-designed late-phase trials.

Consistent with the above, several clinical trials have reported improvements in pain and functional outcomes after intra-articular MSC treatment (Lamo-Espinosa et al., 2021; Kim et al., 2023; Sadri et al., 2023). For example, a trial involving umbilical cord–derived MSCs reported reductions in knee pain and stiffness accompanied by decreases in WOMAC and VAS scores (Matas et al., 2024; Wang et al., 2025). While these findings support symptomatic improvement signals, cross-trial comparisons remain challenging due to differences in eligibility criteria, OA severity, product characterization, dose/frequency, and co-interventions, which may contribute to heterogeneous effect estimates. Umbilical cord–derived MSCs have been investigated in early to moderate OA phenotypes, and adipose-derived MSCs have also shown potential in symptom improvement and modulation of the inflammatory milieu, supported by the accessibility of adipose tissue as a cell source (Mebarki et al., 2021; Pico et al., 2025). Nevertheless, these sources of heterogeneity are also likely contributors to unstable pooled effects and limited reproducibility, thereby reinforcing the translational gap between trial volume and proven efficacy. Claims of disease modification should therefore be made cautiously, given that structural endpoints and long-term durability are not consistently available or uniformly reported across studies.

Despite these encouraging results, safety and side effects remain variable across studies. Existing clinical trial data indicate that intra-articular administration is generally not associated with severe systemic adverse reactions, whereas local transient events such as swelling, pain, and erythema are commonly reported and usually self-limiting (Chen C.-F. et al., 2021; Lamo-Espinosa et al., 2021; Sadri et al., 2023). However, long-term safety remains an area of ongoing research, particularly regarding repeated administrations and cumulative exposure (Wiggers et al., 2021), and short follow-up windows together with incomplete results dissemination may underestimate uncommon or delayed adverse events. Higher doses may trigger transient inflammatory responses that are typically localized and reversible but warrant attention, especially in patients with pre-existing joint conditions (Hwang et al., 2021). In addition, safety inference may be affected by incomplete results reporting and the time lag between trial registration/completion and peer-reviewed publication, which can obscure uncommon or delayed adverse events.

Thus, future studies should systematically evaluate safety across different cell sources and explicitly analyze dose–schedule relationships and administration routes under standardized protocols, with longer follow-up and transparent results dissemination. Importantly, these efforts should be paired with harmonized product characterization and rigorous comparator-controlled designs to determine the reproducibility of efficacy signals and whether benefits consistently reach clinically meaningful thresholds.

### 4.6 Future research directions and challenges

First, multicenter Phase III trials with longer follow-up should be designed to evaluate stem cell–based interventions in larger and more diverse populations and to determine the durability of symptomatic benefit while assessing structural outcomes. This emphasis is necessary because the current evidence base is challenged by heterogeneity in products, dosing schedules, comparators, and outcome timing, and only rigorously controlled late-phase trials with appropriate comparators and sufficient follow-up can convert expanding trial activity into more definitive and generalizable conclusions for clinical practice.

Another key focus is improving and standardizing preparation protocols and delivery methods. Harmonized procedures for collection, culture/processing, and clinical administration are needed to improve comparability and reproducibility across studies, because variability in expansion efficiency, purity, and biological activity can plausibly contribute to unstable clinical signals (Chun et al., 2019; Jin et al., 2023). Optimizing *in vitro* conditions, adopting efficient expansion approaches, and implementing robust quality-control standards may improve consistency across batches and sites (Chen et al., 2024). Importantly, the translational value of standardization lies in reducing cross-trial non-comparability and improving reproducibility rather than simply producing “higher quality” products in an abstract sense.

Similarly, the administration route is likely to influence local exposure and retention, and procedural variability may contribute to heterogeneous outcomes. Evidence suggests that injection approaches can affect distribution and retention of delivered products (Foo et al., 2021). Imaging-guided injections and more standardized procedural protocols may improve delivery accuracy and reduce avoidable variability at the site level (Huang et al., 2020; Ning et al., 2022). Targeted delivery systems and local controlled-release strategies may also address a practical driver of inconsistent efficacy, namely, limited persistence or bioactivity at the disease site (Khayambashi et al., 2021; Tatullo et al., 2022). In parallel, gene-editing and engineered secretory profiles have been proposed to enhance functions relevant to OA (Damasceno et al., 2020; Hassanzadeh et al., 2022), but these approaches also introduce greater manufacturing complexity, higher regulatory thresholds, and added challenges for cross-trial comparability. Therefore, their translational value ultimately requires confirmation through rigorous comparator-controlled studies, standardized reporting, and longer follow-up rather than being inferred from technical feasibility alone.

Finally, precision and data-driven approaches may help improve patient selection and clarify heterogeneity of response, but their clinical utility requires prospective validation. Genomic and bioinformatic strategies may support stratification of biologically appropriate subgroups (Chen B. et al., 2021), and machine-learning methods may aid prediction of response or adverse events (Liu et al., 2020). However, these tools depend on high-quality, shareable datasets and standardized outcome definitions and are vulnerable to bias and limited generalizability when inputs and endpoints are inconsistent across sites (Shende and Devlekar, 2021; Goyal and Malviya, 2023). Consequently, data-driven personalization should be integrated with harmonized endpoints, transparent reporting, and robust trial designs to ensure that predictive performance translates into reliable clinical decisions.

Overall, technological innovation may enable safer and more personalized stem cell–based strategies, but clinically meaningful translation will require standardized manufacturing and reporting, rigorous late-phase trials with appropriate comparators, longer follow-up, and complete results dissemination.

### 4.7 Limitations

This study has several limitations inherent to registry-based landscape analyses. First, although trial registries provide a valuable overview of research activity and design characteristics, registry entries are not equivalent to peer-reviewed evidence and should not be interpreted as direct proof of effectiveness or safety. Information recorded at registration may be incomplete, inconsistently updated, or modified over time, and key fields such as allocation concealment, blinding, comparators, dosing details, and outcomes may be missing or described with variable precision. As a result, misclassification of intervention type, source, dosing regimen, or endpoint category is possible despite careful data extraction. In addition, registry data are susceptible to reporting bias, including selective outcome reporting and the possibility that unfavorable or null findings are less likely to be fully reported or subsequently published. The time lag between trial completion and public dissemination further widens the gap between registered activity and the available peer-reviewed evidence base, which may lead to an overestimation of apparent maturity when judging the field solely by trial counts or completion status. Moreover, because our dataset was derived from specific registry platform, coverage may be incomplete and the identification of duplicate or overlapping registrations across databases can be challenging, which may affect the completeness and comparability of the landscape description.

Second, our analyses were descriptive and based on publicly available registry records, without access to patient-level data, standardized outcome datasets, or complete adverse event reporting; therefore, we could not estimate pooled treatment effects, evaluate dose–response relationships, or formally assess risk of bias and evidence certainty across trials. The substantial clinical and methodological heterogeneity across cell products, manufacturing protocols, dosing schedules, co-interventions, eligibility criteria, and follow-up durations limits cross-study comparability and restrict the inferences that can be drawn from aggregate registry fields. Third, despite broad searching, some trials may not have been captured due to differences in registry coverage, delayed registration, inconsistent terminology, or duplicate registrations across platforms; conversely, some records may represent amendments or overlapping entries that are difficult to identify with certainty. Finally, because registries are continuously updated, our results reflect a time-stamped snapshot of the landscape at the point of data extraction and may not fully represent subsequent registrations, updates, or newly published results.

## 5 Conclusion

Registered clinical trials of stem cell therapy for osteoarthritis indicate active and expanding research and report potential signals of symptom and functional improvement with mostly local, transient short-term adverse events. However, evidence remains heterogeneous, and long-term safety, durability of benefit, and structural modification are still uncertain. Our descriptive registry-based analysis underscores the need for adequately powered, rigorously controlled late-phase trials with standardized protocols, harmonized outcome measures, longer follow-up, and transparent results reporting to determine clinically meaningful efficacy and clarify the role of stem cell–based interventions alongside existing treatments.

## Data Availability

The raw data supporting the conclusions of this article will be made available by the authors, if requested, without undue reservation.
